# A Case of Haemathorax Following an Incised Wound of the Thoracic Wall

**Published:** 1888-10

**Authors:** Norval H. Pierce

**Affiliations:** Chicago


### A CASE OF HÆMOTHORAX FOLLOWING AN INCISED WOUND OF THE THORACIC WALL.*

BY NORVAL H. PIERCE, M. D., CHICAGO.

The patient whom I exhibit to you is 19 years old, of healthy parents, and was six months ago in good health.

On July 13, 1887, at 3:30 p. m., while cutting a piece of wood with the stick adjusted against his chest, he accidentally stabbed himself in the third intercostal space, half an inch to the left of the right parasternal line. The knife, which I hold in my hand, presents a sharp blade two inches in length and half an inch in breadth. After the accident he walked to a neighboring surgery, thinking the wounds of little consequence; but when he arrived the loss of blood externally was sufficient to saturate his clothes and caused syncope.

The doctor prognosticated death in a few hours and summoned the patrol wagon. At 7 p. m., or three and a half hours after the stabbing, I was summoned. He had received no treatment up to this time. I found the man in the condition of one who has sustained a very serious loss of blood—pale features, pinched, eyes sunken, and pupils widely dilated, surface cold and clammy, impaired hearing, pulse nearly imperceptible and very rapid, respiration sighing. There was very slight external bleeding at the wound, which was about an inch in length. From an inch above the sternum to the lower border of the liver on the right side was dull, but at this time the liver was not displaced. No blood was expectorated. I closed the wound antiseptically, ordered an ice-bag for the chest, warm bottles to the lower extremities, head lowered, whisky, half ounce every forty-five minutes, together with ergot, very little liquids, and absolute rest.

For several days after this his life hung in the balance. The

*Read before the Chicago Medical Society, January 3, 1888.

pulse improved gradually, but the anæmia persisted, dyspnœa arose, together with great restlessness and troublesome insomnia, and the right pleural cavity remained flat on percussion, the lower border of the liver gradually descended and the heart was displaced to the left, indicating that the hæmorrhage was slowly progressing. At this time the advisability of enlarging the wound and taking up the bleeding vessel, or searing the lung with Paquelin's cautery, was entertained. But such a procedure entailed great risk and no small amount of uncertainty, the location of the bleeding point being unsettled. These facts, together with that of the patient holding his own, even in a low condition, deterred operating.

Because of increasing dyspnœa I consulted with Dr. Fenger as to the advisability of aspirating, under the existing circumstances, and on July 27 a gallon of dark-colored blood was drawn away. This did not cause the dullness over the right thoracic area to disappear, but relieved the difficult breathing and lessened the restlessness and insomnia.

On August 20 I again aspirated, the lower border of the liver being within an inch of the iliac crest. Another gallon was drawn away. Under the microscope the white corpuscles were not greatly increased. Up to this time the temperature had ranged from 100° to 101° F. Afterwards it fell to normal once or twice, but generally the thermometer registered 100° F.

On September 5 diarrhœa set in and lasted two or three days. Cough developed soon after, with very offensive expectoration. The liver dullness was gradually descending and extreme dyspnœa began. As the fluid in the pleural cavity now contained a marked increase in white corpuscles, I decided to perform the radical operation of empyema. On September 23 this was attempted. At this time there was a marked lowering of his general condition. At the time of the incision I purposed removing one or more ribs, but on account of the alarming manner in which he took the ether, we thought it best to be as expeditious as possible. The tissues of the seventh intercostal were therefore simply incised, about a gallon of fluid allowed to escape, and the opening plugged with iodoform gauze. At the end the patient was in a state of collapse, brought on, I believe, by the too sudden escape of the fluid. He rallied, however, and the next day a tube was inserted and the cavity washed with a saturated solution of boracic acid at 100° F., which was repeated every second day thereafter. Fever and cough disappeared on the second day. The washings grew less and less colored, and in a few days the tube was removed.

The case sets forth not so much the innate goodness of conservative surgery as it does the great tolerance of the thoracic cavity of effusions nearly if not quite equal to that of the abdomen. It also shows what a great quantity of blood may be lost without death, over three gallons being taken away in as many tappings. It likewise shows that the wound from which such a hæmorrhage could issue is capable of spontaneous arrest.

As regards the place for the incision, I would say the seventh intercostal space is too low, as after the first week the movements of the diaphragm interfered with the drainage tube.

As you may see, this slight cut an inch in length, with its attendant disasters, has left its mark. The right shoulder is depressed one inch. The external circumference of the right side is less than that of the left.

